# Compromised Well-being: A Cross-Sectional Study of Quality of Life Among Healthcare Staff in Sousse, Tunisia

**DOI:** 10.1192/j.eurpsy.2026.11530

**Published:** 2026-07-31

**Authors:** R. Ghammam, H. Kalboussi, O. K. Ben Saad, S. Ben Fredj, N. Zammit, A. Skhiri, F. Chouikha, A. Erguez, R. Akrimi, A. Chouchene, I. Harrabi, M. Jihene

**Affiliations:** 1 Université de Sousse, Faculté de Médecine de Sousse; 2 Hôpital Universitaire Farhat hached, Service de Médecine Préventive, LR19SP03; 3 Hopital Universitare Farhat Hached, Service de Médecine Préventive, LR19SP03; 4 Hôpital Universitaire Farahat Hached, Service de Médecine de Travail, LR19SP03; 5 Médecine Préventive et Communautaire, Hopital Universitaire Farhat hached; 6 Hopital Universitaire Farhat hached, Laboratoire de Biochimie; 7 Médecine de Famille, Université de Sousse, Faculté de Médecine de Sousse; 8 Hopital Universitare Farhat Hached, Service de Médecine de Travail, LR19SP03, Sousse, Tunisia

## Abstract

**Introduction:**

The quality of life for healthcare professionals is a key determinant of both personal well-being and the quality of patient care. Healthcare workers face immense occupational stress, long hours, and high emotional demands. These stressors can significantly compromise their physical and mental health, leading to burnout and reduced professional satisfaction.

**Objectives:**

The quality of life for healthcare professionals is a key determinant of both personal well-being and the quality of patient care. Healthcare workers face immense occupational stress, long hours, and high emotional demands. These stressors can significantly compromise their physical and mental health, leading to burnout and reduced professional satisfaction.

**Methods:**

We performed a cross-sectional study to evaluate the quality of life among healthcare professionals at Farhat Hached University Hospital in Sousse, Tunisia. A simple random sample of staff was selected to ensure representativeness. All participants were provided with an information sheet and gave their informed consent before taking part. Data were collected via a pretested, self-administered questionnaire. Quality of life (QoL) was measured using the French version of the 12-Item Short Form Health Survey (SF-12) that contribute to two distinct scores: the Physical Quality of life (PQoL) and the Mental Quality of life (**MQoL**)

**Results:**

Our study included 205 healthcare professionals, of whom 89.3% were female. The mean age of the participants was 43.57±8.28 years, and the largest professional groups were nurses (42.4%) and health technicians (38%).

We found a high prevalence of compromised quality of life. Specifically, **71.2%** of respondents reported a compromised physical quality of life (PQoL), with a mean score of 43.50±8.97 (min=18.68, max=61.96). Moreover, a compromised mental quality of life (MQoL) was observed in **80.5%** of the healthcare professionals, with a mean score of 40.50±9.14 (min=18.63, max=64.19).

When comparing professional groups, we found no significant difference in the prevalence of compromised PQoL between doctors (59.5%) and nurses and technicians (73.8%, p=0.081). Similarly, the prevalence of compromised MQoL was comparable between doctors (81.1%) and nurses/technicians (80.4%, p=0.920).

**Conclusions:**

Results of our study demonstrate a significant burden on the well-being of healthcare professionals, highlighting a critically compromised level of both physical and mental quality of life. This compromised state is likely attributable to the intense workload and inherent stressors of the healthcare environment, posing a risk not only to the health of the staff but also to the quality and safety of the care they provide. Therefore, we urge the implementation of targeted interventions designed to improve the quality of work life.

**Disclosure of Interest:**

None Declared

